# Co_3_O_4_-Based Materials as Potential Catalysts for Methane Detection in Catalytic Gas Sensors †

**DOI:** 10.3390/s24082599

**Published:** 2024-04-18

**Authors:** Olena Yurchenko, Patrick Diehle, Frank Altmann, Katrin Schmitt, Jürgen Wöllenstein

**Affiliations:** 1 Fraunhofer Institute for Physical Measurement Techniques (IPM), 79110 Freiburg, Germany; katrin.schmitt@ipm.fraunhofer.de (K.S.); juergen.woellenstein@ipm.fraunhofer.de (J.W.); 2 Fraunhofer Institute for Microstructure of Materials and Systems (IMWS), 06120 Halle, Germany; patrick.diehle@imws.fraunhofer.de (P.D.); frank.altmann@imws.fraunhofer.de (F.A.); 3 Department of Microsystems Engineering (IMTEK), University of Freiburg, 79110 Freiburg, Germany

**Keywords:** cobalt oxide, catalyst, catalytic sensors, morphology

## Abstract

The present work deals with the development of Co_3_O_4_-based catalysts for potential application in catalytic gas sensors for methane (CH_4_) detection. Among the transition-metal oxide catalysts, Co_3_O_4_ exhibits the highest activity in catalytic combustion. Doping Co_3_O_4_ with another metal can further improve its catalytic performance. Despite their promising properties, Co_3_O_4_ materials have rarely been tested for use in catalytic gas sensors. In our study, the influence of catalyst morphology and Ni doping on the catalytic activity and thermal stability of Co_3_O_4_-based catalysts was analyzed by differential calorimetry by measuring the thermal response to 1% CH_4_. The morphology of two Co_3_O_4_ catalysts and two Ni_x_Co_3−x_O_4_ with a Ni:Co molar ratio of 1:2 and 1:5 was studied using scanning transmission electron microscopy and energy dispersive X-ray analysis. The catalysts were synthesized by (co)precipitation with KOH solution. The investigations showed that Ni doping can improve the catalytic activity of Co_3_O_4_ catalysts. The thermal response of Ni-doped catalysts was increased by more than 20% at 400 °C and 450 °C compared to one of the studied Co_3_O_4_ oxides. However, the thermal response of the other Co_3_O_4_ was even higher than that of Ni_x_Co_3−x_O_4_ catalysts (8% at 400 °C). Furthermore, the modification of Co_3_O_4_ with Ni simultaneously brings stability problems at higher operating temperatures (≥400 °C) due to the observed inhomogeneous Ni distribution in the structure of Ni_x_Co_3−x_O_4_. In particular, the Ni_x_Co_3−x_O_4_ with high Ni content (Ni:Co ratio 1:2) showed apparent NiO separation and thus a strong decrease in thermal response of 8% after 24 h of heat treatment at 400 °C. The reaction of the Co_3_O_4_ catalysts remained quite stable. Therefore, controlling the structure and morphology of Co_3_O_4_ achieved more promising results, demonstrating its applicability as a catalyst for gas sensing.

## 1. Introduction

Catalytic gas sensors, known as pellistors, are commercially available sensors used to detect flammable gases and to safely monitor gas concentrations below the lower explosive limit (LEL) [1,2,3,4]. The main area of application for pellistors is the identification of explosion risk, which arises if the concentration of flammable gases exceeds the lower explosive limit [2]. Especially light alkanes such as methane, propane, and butane are of particular interest for safety monitoring. Methane is an essential gas for various technologies, and as a main component of natural gas, it is still widely used as energy source in industrial applications and homes [5,6,7]. Propane and butane are the components of liquefied petroleum gas (LPG) commonly used in heating systems, cooking appliances, and vehicles [8]. Gas leaks in the home are extremely dangerous for people and pose a high risk of property damage [9].

Pellistors were first described by Alan Baker in the early 1960s [10]. A pellistor consists of two sensor elements: the active sensor coated with a catalyst layer and the passive sensor element without any catalyst. The passive sensor only responds to the changes in environmental conditions, such as humidity and temperature, while the active sensor interacts with target gases. Because of these interactions, the target gases are oxidized, and heat evolves, which is detected as thermal response of the pellistor. To activate catalytic oxidation of target gases, the sensor is operated at a specified temperature [11,12]. 

The oxidation of methane requires high operation temperatures (≥450 °C) due to high activation energy caused by the high stability of its C–H bonds [13,14]. However, high operation temperatures accelerate the aging and degradation of the catalysts. Propane and butane, as well as other flammable gases, are more reactive gases that are oxidized at much lower operation temperatures than methane and are therefore easier to detect [1]. Therefore, the focus in development of new catalysts for catalytic gas sensors is on suitable materials for detecting methane. Aluminum oxide-based catalysts containing a large amount of catalytically highly active Pd and Pt metals are used in commercially available pellistors to ensure the proper detection of gases, primarily of inert methane. The high metal content is used to reduce operating temperature and ensure the required sensor life. This is mainly due to the strong tendency of Pd-based catalysts to deactivate during operation. In view of the scarcity of precious metals, these catalysts should be substituted by metal-oxide catalysts containing none or only a low loading of noble metals. Metal oxides from transition metals (Co, Mn, Fe, Cr, Ni, Cu, etc.) with perovskite or spinel structure as well as transition metal-substituted hexa-aluminates are considered as alternative candidates to noble metal-based catalysts for oxidation of light hydrocarbons, mainly methane, at lean conditions [7,15,16,17,18,19]. Low concentrations applied at lean conditions in catalysis prevail also in gas sensing for safety monitoring, thus the target concentration range (the LEL range) for methane detection is between 0.2% and 4%. Transition metal oxides offer an advantage over catalytically inert alumina due to their intrinsic catalytic activity. Their catalytic activity is attributed to the multiple oxidation states (e.g., Co^3+^/Co^2+^, Fe^2+^/Fe^2+^, Cu^2+^/Cu^+^, and Mn^4+^/Mn^3+^/Mn^2+^), lattice defects (i.e., oxygen vacancies), high oxygen storage capacity, and oxygen transfer capability [19]. The multiple oxidation states in the crystal structures are considered as active sites for adsorption and activation of reactant molecules and oxygen [19]. High oxygen storage capacity and oxygen transfer capability help to restore the surface-adsorbed oxygen species consumed through the catalytic oxidation by release of lattice oxygen species and formation of oxygen vacancies.

Co_3_O_4_-based materials are the most promising catalysts for oxidation of methane among all transition metal oxides. Co_3_O_4_ possesses a spinel-type structure with two oxidation states (Co^2+^/Co^3+^). The easy reduction of Co^3+^ to Co^2+^ in Co_3_O_4_ facilitates the formation of oxygen vacancies as surface defects at low temperatures, promoting oxygen mobility. High oxygen mobility and highly active surface oxygen species of Co_3_O_4_, whose formation is supported by oxygen vacancies, contribute to the activation and breaking of the C–H bond at low temperature [14,15,17,20,21,22]. In addition, the dissociation of the C–H bond is the rate-controlling step during oxidation of saturated hydrocarbons. The specific electronic structure of Co_3_O_4_ with partially filled d-orbitals of Co^3+^ and Co^2+^ (d^6^ and d^7^, respectively) lead to the reduction of the activation energy for methane dissociation via direct interaction of C–H orbitals with d-type orbitals of Co cations [23]. 

Moreover, the incorporation of hetero atoms such as Ce, Ni, Cr, etc., into the spinel structure of Co_3_O_4_ can improve further its catalytic ability. Heteroatoms induce lattice distortion, which could increase the amount of active surface oxygen species and generate more structural defects (e.g., oxygen vacancies) [24]. On the other hand, doping of Co_3_O_4_ with another metallic element can improve its textural properties [19]. Especially, cobalt–nickel mixed oxides, i.e., Ni_x_Co_3−x_O_4_, effectively reduce oxygen vacancy formation energies compared to pure Co_3_O_4_, thus increasing catalytic activity [21,22,25,26]. However, researchers have disagreed on which nickel to cobalt molar ratio (Ni:Co) exhibits the best catalytic performance in methane oxidation. NiCo_2_O_4_ with the high Ni:Co ratio of 1:2 was found by many authors to be the most active catalyst compared to other Ni_x_Co_3−x_O_4_ or pure oxides [15,16,21]. Yet, other authors have reported reduced catalyst stability at temperatures > 400 °C because of sintering and partial decomposition to NiO accompanied with performance degradation when the Ni:Co ratio is high. Reducing the Ni:Co ratio can slow down thermal deactivation of Ni_x_Co_3−x_O_4_ catalysts, improving their long-term stability at higher operation temperatures (450 °C) [27,28].

However, it was demonstrated by several studies on pure Co_3_O_4_ catalysts that morphology and textural properties also play a decisive role in catalytic activity due to variations in the porosity, crystal structure, formation of surface defects, and surface-active species [14,16,28,29,30,31]. In general, the surface atomic configurations and surface defects of catalyst particles can change the adsorption and desorption properties (e.g., surface oxygen bond strength) influencing the catalytic activity of the surfaces [23]. Furthermore, morphology can strongly influence the stability of catalysts. Lyu at al. reported that three-dimensionally ordered, mesoporous Co_3_O_4_ is not appropriate for application in pellistors due to the high instability of its structure under pellistor operation conditions. Especially, the mesoporous Co_3_O_4_ catalyst functionalized with Au-Pd nanoparticles exhibited strong deactivation because of strong metal oxide sintering [32]. Thus, the suitable structure and morphology of the metal oxide had a crucial impact on performance of the catalytic sensors. Despite the high interest in metal oxides as catalysts for catalytic combustion, metal oxide materials have hardly been tested for use in catalytic gas sensors [32,33]. Although the catalytic oxidation of target gases underlies the sensor’s response, the results of standard catalyst studies using reactors cannot be easily transferred to catalytic gas sensors because the catalysts are prepared for examination according to the requirements for the catalytic bed reactors, e.g., by dilution with other components, pelletization, etc. [18]. Moreover, not all available catalyst morphologies are suitable for application in sensors. For example, hierarchical morphologies and micrometer-sized particles are not appropriate for sensors. In addition, the thermal conductivity of the catalyst layer largely determines the thermal response of the catalytic gas sensor. Therefore, the catalyst morphology is extremely important for achieving optimal thermal conductivity, and potential catalysts for catalytic gas sensors should be examined under appropriate conditions.

In the present work, we investigated the impact of the doping of Co_3_O_4_ with Ni on the catalyst’s morphology and catalytic response towards lean methane (1 vol%) along with its short-term stability under 400 °C and 450 °C. Two pure Co_3_O_4_ and two Ni-modified cobalt oxide catalysts, i.e., Ni_x_Co_3−x_O_4_, with two different Ni to Co ratios (1:2 and 1:5), were synthetized by co-precipitation techniques commonly used for the synthesis of pure and heterogeneous metal oxide catalysts due to its simple preparation procedure and easy scaling-up in industrial production [21,26,30]. The given Ni to Co ratios were chosen to examine the effect of Ni quantity on the thermal response and stability of Co_3_O_4_ catalysts because, according to literature, the catalysts with a 1:2 ratio showed mostly the highest activity, and those with a 1:5 ratio had improved stability [16,28]. In the case of Co_3_O_4_, N_2_ or air was used during synthesis to obtain particles of different morphology according to [16], which reported on the atmosphere having a key role in controlling the particle morphology of Ni_x_Co_3−x_O_4_ catalysts. 

The thermal response of the catalysts was measured in the temperature range pertaining to pellistor applications by means of differential scanning calorimetry (DSC). In DSC, the thermal signal related to the heat evolved by methane oxidation is measured. Details concerning the DSC method and the procedure adapted for catalysts examinations can be found in our recent publication [34]. Previous investigations on one of the examined catalysts (NiCo_2_O_4_) confirmed that DSC measurements can be used to check the catalytic activity of potential catalysts for pellistors [35]. 

## 2. Materials and Methods

Pure (mCo_3_O_4_ and sCo_3_O_4_) and Ni-doped Co_3_O_4_ (NiCo_2_O_4_ with Ni:Co ratio of 1:2; Ni_0.5_Co_2.5_O_4_ with Ni:Co ratio of 1:5) catalysts were synthesized by the same precipitating procedure as described in [21]. mCo_3_O_4_ was synthesized in N_2_ atmosphere, as reported in the original publication, while sCo_3_O_4_ was prepared in air. In the case of NiCo_2_O_4_ and Ni_0.5_Co_2.5_O_4_, N_2_ atmosphere was applied. Furthermore, a part of the Co precursor was substituted by a Ni precursor (Ni(NO_3_)_2_ 6 H_2_O) in order to obtain the specific Ni:Co ratio (1:2 or 1:5). The composition of the final NiCo_2_O_4_ catalyst was examined by energy dispersive X-ray analysis. The mean Ni:Co ratio in NiCo_2_O_4_ corresponded to the ratio used in the synthesis (1:2). 

The mCo_3_O_4_ sample was synthesized by the following precipitating procedure: Co(NO_3_)_2_ 6H_2_O (17.46 g; Carl Roth, Karlsruhe, Germany; >98%) was dissolved in 100 mL deionized water at 23 °C. Then, a KOH solution (1 mol L^−1^, 300 mL) was added under the bubbling of nitrogen gas and continuous, strong stirring. A precipitate was collected and washed three times with hot, deionized water (60 °C), followed by drying at 130 °C for 24 h. The obtained solid was ground to powder in a mortar and further calcined at 350 °C in air for 24 h, forming the synthesized catalyst, which was denoted as mCo_3_O_4_. 

To examine the effect of calcination temperature on the catalytic activity, a part of sCo_3_O_4_ was investigated directly after drying at 130 °C for 24 h (denoted as nc_sCo_3_O_4_), and a part was additionally calcined at 400 °C for 24 h (denoted as nc_sCo_3_O_4_ 24 h 400 °C). 

A scanning transmission electron microscope (STEM) equipped with an in-lens secondary electron detector (SE) and an energy dispersive X-ray (EDX) detector (Hitachi HF 5000, Hitachi, Tokyo, Japan) was used to visualize the morphology and to analyze the composition of the catalysts. The SE detector gives access to surface information and visualizes the surface topography of the sample. In the following, the images are described as SE-STEM since the images were acquired in STEM mode, although the electrons are not transmitting the sample. 

Crystalline structure and phases of catalysts were checked by means of X-ray diffraction (XRD) analyses (Empyrean, Malvern Panalytical Ltd., Malvern, UK) using Cu Kα radiation.

Differential scanning calorimetry (DSC) (STA 409 CD-QMS 403/5 SKIMMER, Netzsch, Selb, Germany) was used to examine the thermal response of the catalysts to 1% methane in dry air. The investigations using DSC can only be carried out method specifically in a dry gas atmosphere. DSC is, in its principle, very similar to pellistor measurements. In DSC, the temperature difference between the empty reference pan and the sample pan filled with a powder is detected at increasing temperature (dynamic conditions) as an electrical signal normalized to the sample weight (μV mg^−1^). 

To investigate catalytic activity, the measurement procedure applied for standard measurements was adapted [34]. In our experiments, the same procedure was used as for testing the gas sensors. The temperature difference between sample and reference pan was measured at isothermal conditions, when first, dry compressed air was introduced into the system to record a baseline and then 1% methane (1 vol% CH_4_ in synthetic air, Air Liquide, Düsseldorf, Germany). At each temperature level, the baseline recorded in air was set to zero [34]. Figure S1 (Supplementary Materials) shows an exemplary DSC signal obtained for sCo_3_O_4_ catalyst upon exposure to 1% methane (orange bars) at a predefined temperature program (red line). The height of the signal was considered for catalyst characterization. The dynamic behavior, such as response or recovery time, was not analyzed since it depends on the instrumentation or sensor used and does not provide any valuable information in this case.

The measured DSC voltage corresponds to the heat produced during catalytic oxidation. The experiments were performed on 8 g catalyst using an aluminum pan at a constant gas flow rate of 100 mL min^−1^. To investigate the thermal stability, samples were held in compressed dry air containing 400 ppm CO_2_ at 400 °C or 450 °C for 24 h and 12 h, respectively. Air was chosen instead of synthetic air to achieve conditions more realistic for sensor applications. The catalytic activity was measured before and after thermal treatment of samples using the same temperature profile. 

## 3. Results and Discussion

### 3.1. Structure and Morphology of Investigated Catalysts

**Figure 1:**
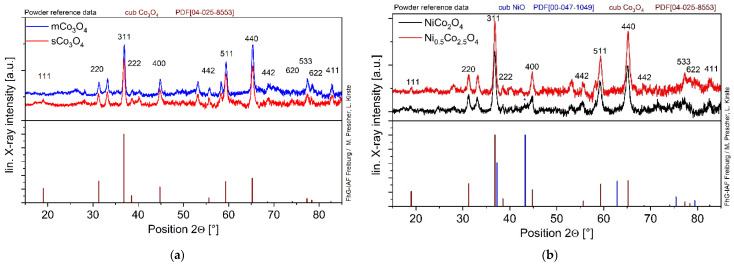
XRD patterns of (**a**) undoped mCo_3_O_4_ and sCo_3_O_4_ oxides as well as (**b**) Ni-doped oxides NiCo_2_O_4_ and Ni_0.5_Co_2.5_O_4_; the reference data are listed below.

The X-ray diffraction patterns of undoped and Ni-doped Co_3_O_4_ oxides are shown in Figure 1. The catalysts are fine crystalline with small crystallites or many defects in crystallites, which is visible from the line broadening of XRD diffraction peaks. The main peaks can be assigned to the cubic Co_3_O_4_ spinel structure (No. 04-025-8553). No peaks related to Co(OH)_2_ or partially oxidized CoO(OH) were observed in the XRD patterns of Co_3_O_4_ (Figure 1a), indicating the complete transformation of Co(OH)_2_ to Co_3_O_4_. However, XRD patterns of all catalysts contain some peaks, e.g., at 2Θ = 33.3°, 40.3°, 53.2°, and 58.4°, which cannot be assigned to any crystalline phase. No mentions of these additional peaks were found in the literature, although the crystallinity of catalysts in the literature is significantly higher. The patterns of mCo_3_O_4_ and sCo_3_O_4_ catalysts reveal minor variations in the crystal structure apart from the peak at 2Θ = 19.0° assigned to the (111) plane, which is absent in mCo_3_O_4_. DRIFT and DFT calculations revealed that the (111)-plane of Co_3_O_4_ is responsible for the facile activation of the C–H bond and its high activity [18]. X-ray diffraction of Ni-doped Co_3_O_4_ oxides (Figure 1b) showed that both Ni_x_Co_3−x_O_4_ catalysts have the same spinel structure as Co_3_O_4_. That coincides with the results from Tao et al. [21], who reported that Ni_x_Co_3−x_O_4_ catalysts synthesized by co-precipitation with KOH exhibit the same diffraction pattern as Co_3_O_4_. This reveals the integration of Ni cations into the crystallographic lattice of spinel Co_3_O_4_. However, in contrast to the literature, some peaks assigned to NiO ((200) at 2Θ = 43.3° and (220) at 2Θ = 62.9°) were found in the NiCo_2_O_4_ catalyst. Additionally, the lattice planes (442), (533), and (622) of Co_3_O_4_ are not well formed in NiCo_2_O_4_.

**Figure 2:**
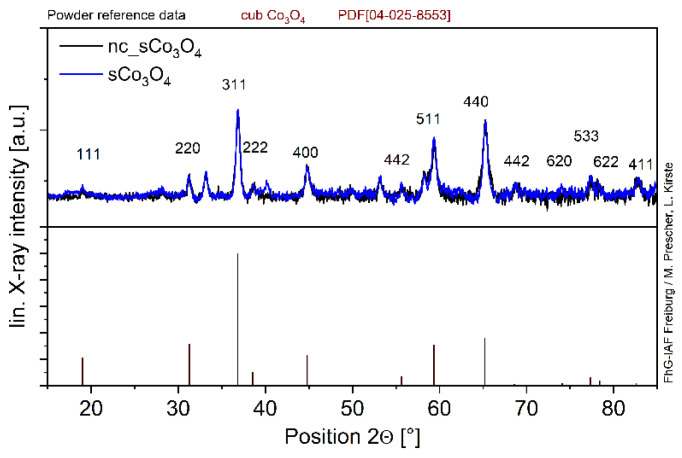
XRD patterns of the dried and calcined sCo_3_O_4_ oxides in comparison; the reference data are listed below.

The dried, non-calcined_sCo_3_O_4_ and calcined sCo_3_O_4_ oxides (Figure 2) exhibit similar XRD patterns revealing that the conversion of Co(OH)_2_ into Co_3_O_4_ was mostly completed after catalyst drying at 130 °C. After calcination at 350 °C, only one additional peak appears at 2Θ = 40.1°. Thus, most alterations happening during the calcination step concern the catalyst surface, such as surface defects and oxygen species.

**Figure 3:**
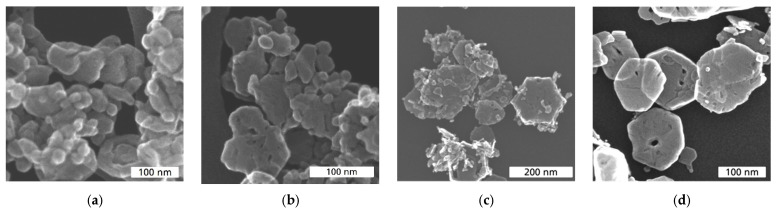
SE-STEM images of (**a**) mCo_3_O_4_; (**b**) sCo_3_O_4_; (**c**) NiCo_2_O_4_; (**d**) Ni_0.5_Co_2.5_O_4_ metal-oxides.

**Figure 4:**
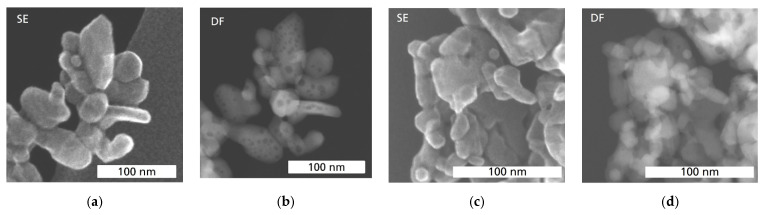
SE-STEM (**a**,**c**) and HAADF-STEM (**b**,**d**) images in comparison with mCo_3_O_4_ (**a**,**b**) and sCo_3_O_4_ (**c**,**d**).

In Figure 3, the morphologies of Co_3_O_4_ and Ni-doped Ni_x_Co_3−x_O_4_ catalysts are shown. mCo_3_O_4_ and sCo_3_O_4_ demonstrate similar morphology with small differences. mCo_3_O_4_ (Figure 3a) reveals rather angular particles of irregular size and shape, while for sCo_3_O_4_ (Figure 3b), particles with sheet morphology are present in addition to small nanoparticles. Moreover, mCo_3_O_4_ particles contain a high number of holes inside (Figure 4a,b), significantly more than sCo_3_O_4_ particles (Figure 4c,d), as the comparison of the corresponding HAADF-STEM images clearly shows. Thus, the morphological investigations indicate that mCo_3_O_4_ and sCo_3_O_4_ materials have different particle morphology and structure, so differences in their interactions with reactants and heat transport are expected. 

**Figure 5:**
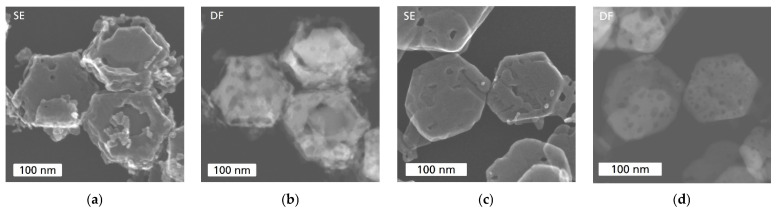
SE-STEM (**a**,**c**) and HAADF-STEM (**b**,**d**) images in comparison with NiCo_2_O_4_ (**a**,**b**) and Ni_0.5_Co_2.5_O_4_ (**c**,**d**).

Both Ni_x_Co_3−x_O_4_ catalysts exhibit a hexagonal sheet morphology (Figure 3c,d), which differs from the morphology of pure Co_3_O_4_ catalysts (Figure 3a,b). Such a morphology is beneficial for the catalysis due to high macroporosity and better gas transport inside the catalyst layer. However, the sheet’s quality of NiCo_2_O_4_ catalyst is very low. Moreover, NiCo_2_O_4_ (Figure 3c) reveals in addition to the defective hexagonal sheets an accumulation of small particles and debris. A high number of diverse defects in the structure of NiCo_2_O_4_ is well visible in HAADF-STEM images (compare Figure 5a,b). In contrast to that, well-formed nanoplatelets of hexagonal shape with a porous surface and holes inside can be observed for Ni_0.5_Co_2.5_O_4_ (Figure 3d and Figure 5c,d).

**Figure 6:**
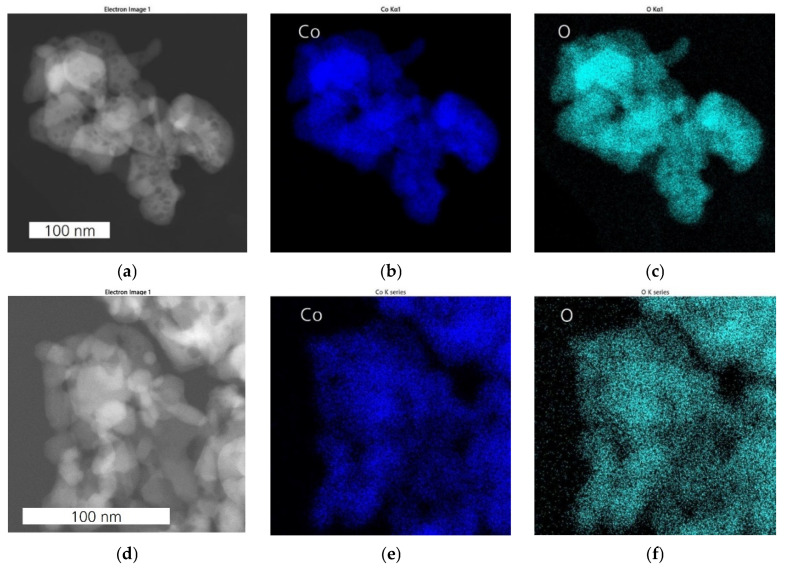
EDX analysis of Co_3_O_4_ catalysts: HAADF-STEM images of mCo_3_O_4_ (**a**) and sCo_3_O_4_ (**d**); the corresponding elemental distribution maps of Co (**b**,**e**) and O (**c**,**f**) in respective metal-oxides.

The EDX analysis of mCo_3_O_4_ (Figure 6a–c) and sCo_3_O_4_ (Figure 6d–f) catalysts shows that the elemental distributions of Co and O coincides, and hence, no segregation was observed. Moreover, no further elements were detected, excluding impurities.

**Figure 7:**
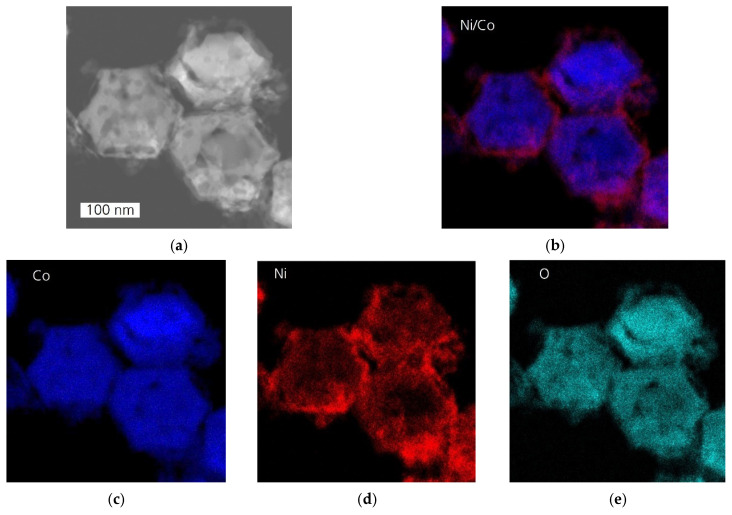
EDX analysis of NiCo_2_O_4_ catalyst: (**a**) HAADF-STEM image; (**b**) overlay of the elemental distribution maps of Ni and Co; the elemental distribution maps of Co (**c**), Ni (**d**), and O (**e**).

EDX elemental mapping of NiCo_2_O_4_ catalyst (Figure 7) demonstrates that Co and O are distributed evenly throughout the nanoplates, whereas Ni is predominantly concentrated at the edge of the hexagonal plates as well as in the nanoparticles and debris. Less nickel is found at the center of the hexagonal plates, revealing strongly pronounced phase separation of Ni as NiO nanoparticles. The phase segregation of NiO and a high number of debris indicate that an excess of Ni disrupts the Ni integration into the Co_3_O_4_ lattice. The investigations reveal that NiCo_2_O_4_ contains a mixture of two metal oxides: the mixed oxide Ni_x_Co_3−x_O_4_ with variable Ni:Co content and the pure NiO.

**Figure 8:**
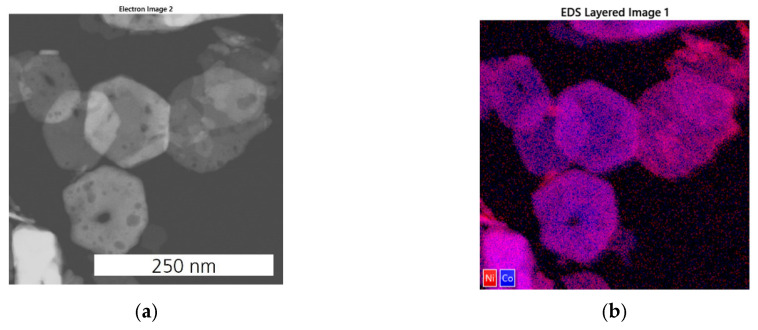

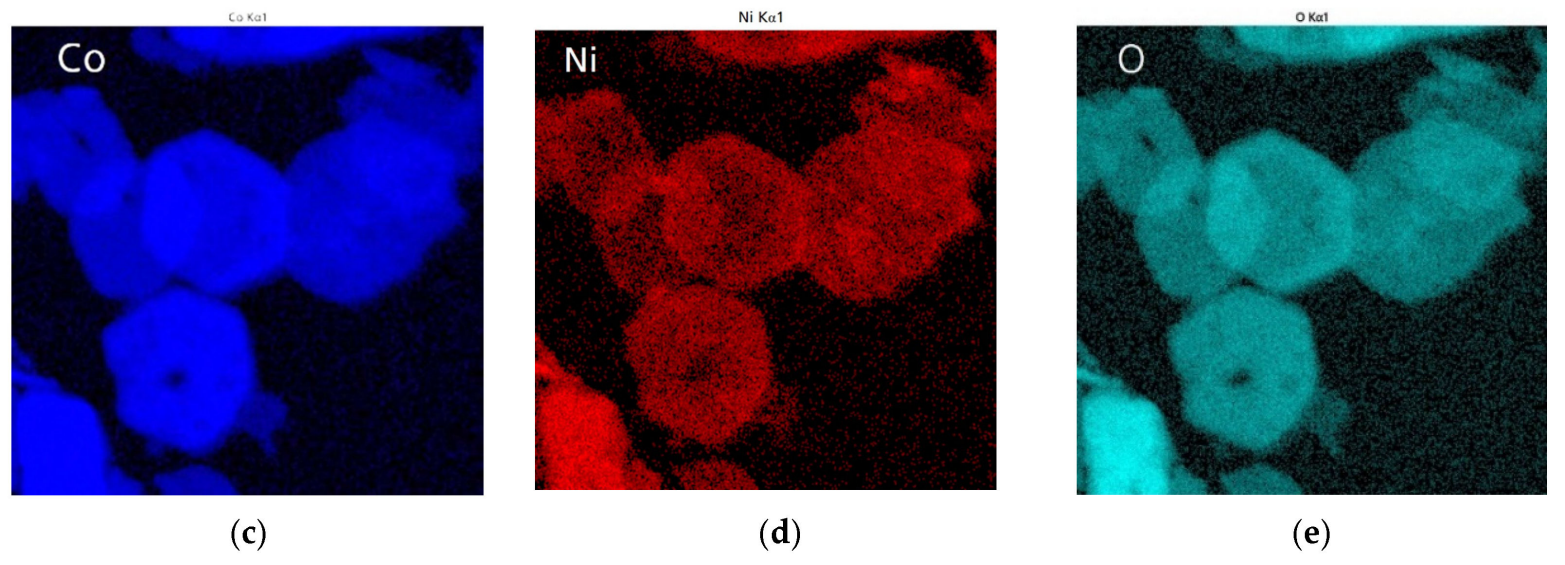
EDX analysis of Ni_0.5_Co_2.5_O_4_ catalyst: (**a**) HAADF-STEM image; (**b**) overlay of the elemental distribution maps of Ni and Co; the elemental distribution of Co (**c**), Ni (**d**), and O (**e**).

EDX elemental mapping of Ni_0.5_Co_2.5_O_4_ oxide indicates that the distribution of Co, O, and Ni is mainly uniform inside hexagonal plates (Figure 8). Thus, Ni is well integrated into the structure of Co_3_O_4_ oxide. Consequently, the application of a lower Ni:Co ratio results in a doped spinel structure, i.e., Ni_x_Co_3−x_O_4_, with highly uniform morphology and composition. However, for some particles, a slight inhomogeneous distribution of nickel in the hexagonal plates is still observed.

### 3.2. Characterization of Catalytic Response

**Figure 9:**
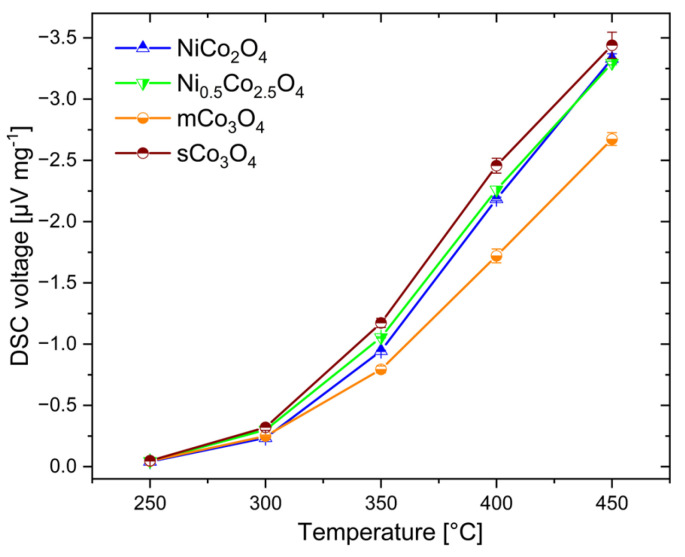
Temperature-dependent DSC response of the four investigated catalysts to 1% CH_4_, with error bars giving the standard deviation from three measurements.

Figure 9 shows the results of DSC measurements on the four catalysts obtained in temperature range between 250 °C and 450 °C with 1 vol% methane. As expected, the DSC voltage increases with higher temperature. At 250 °C and 300 °C, the DSC voltages were comparably low for all catalysts (0.25–0.32 μV mg^−1^ at 300 °C), caused by the low activity of metal oxide catalysts at such low temperatures. Only when the temperature increases above 300 °C does the difference in the catalyst activity become substantial. The Ni-modified catalysts, namely NiCo_2_O_4_ and Ni_0.5_Co_2.5_O_4_, exhibit clearly higher DSC signals than pure mCo_3_O_4_, particularly at 450 °C (3.3 μV mg^−1^ vs. 2.7 μV mg^−1^), which is in agreement with the literature reports about the higher activity of Ni_x_Co_3−x_O_4_ catalysts compared to pure Co_3_O_4_ [16,27]. The differences in activity between NiCo_2_O_4_ and Ni_0.5_Co_2.5_O_4_ are, however, not significant. The higher Ni ratio in NiCo_2_O_4_ does not cause the expected increase in catalytic activity. This can be explained by the segregation of NiO, which exhibits a much lower activity than Ni_x_Co_3−x_O_4_ and Co_3_O_4_ [16,28]. 

Interestingly, sCo_3_O_4_, precipitated in air, seems to slightly outperform the activity of the Ni-modified oxides NiCo_2_O_4_ and Ni_0.5_Co_2.5_O_4_ at all temperatures. The considerable differences in response for mCo_3_O_4_ and sCo_3_O_4_ must be caused by their different morphology and structure, as seen in Figure 3a,b and Figure 4 [29,36]. Obviously, the high structural defect density of mCo_3_O_4_ (Figure 2b) does not improve the catalytic activity of Co_3_O_4_, whereas the sheet morphology of sCo_3_O_4_ seems to have a positive impact on its activity. It is notable that sCo_3_O_4_ exhibits a higher variance in activity compared to mCo_3_O_4_, which correlates with the variations in particle morphology observed for sCo_3_O_4_.

**Figure 10:**
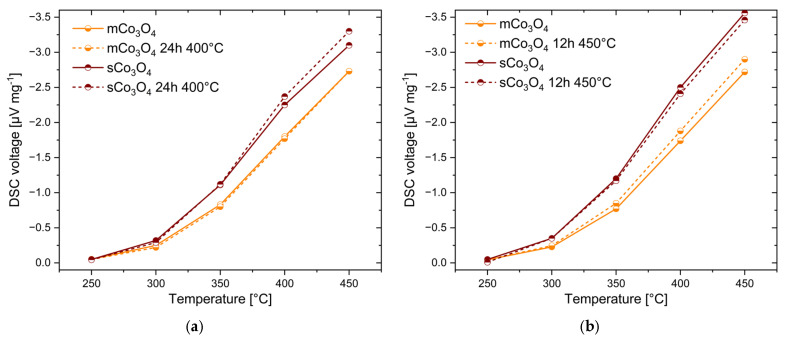
Results of the stability investigations: temperature-dependent DSC response of two Co_3_O_4_ catalysts to 1% methane measured before and after treatment at 400 °C (**a**) and at 450 °C (**b**).

Figure 10 shows the activity of the two Co_3_O_4_ catalysts before and after the treatment in pressurized air at 400 and 450 °C for 24 and 12 h, respectively. mCo_3_O_4_ and sCo_3_O_4_ show either no or a slight decrease or even an increase in the DSC signal after the treatment, revealing their stability under test conditions. The increase in the thermal response after heat treatment can be explained by an improved contact between individual particles in the layer, causing better thermal conductivity and thus better transfer of reaction heat to thermopiles of the detector. 

Regarding XRD and morphology investigations, it is likely that the significantly improved thermal response of sCo_3_O_4_ compared to mCo_3_O_4_ is caused by low defect density and sheet morphology. It is also possible that the (111) lattice plane present in sCo_3_O_4_ contributes to its improved catalytic activity compared to mCo_3_O_4_.

**Figure 11:**
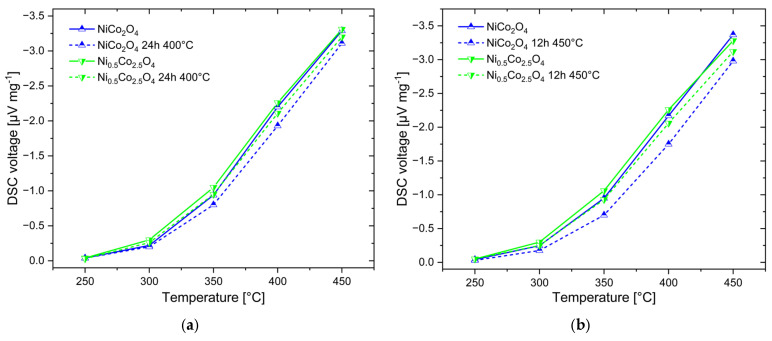
Results of the stability investigations: temperature-dependent DSC response of two Ni_x_Co_3−x_O_4_ catalysts to 1% methane measured before and after treatment at 400 °C (**a**) and at 450 °C (**b**).

In contrast to Co_3_O_4_, NiCo_2_O_4_ and Ni_0.5_Co_2.5_O_4_ (Figure 11) show a reduced DSC signal as a result of the heat treatment. Particularly, the NiCo_2_O_4_ catalyst experiences a considerable decrease in catalytic activity already after a short treatment at both temperatures. The most pronounced decrease from 3.4 μV mg^−1^ to 3.0 μV mg^−1^ was observed for NiCo_2_O_4_ at 450 °C after treatment at 450 °C (Figure 11b). After thermal treatment at 400 °C (Figure 11a), the DSC signal decrease is lower, although it still remains high (difference of 0.3 μV mg^−1^ at 400 °C). Ni_0.5_Co_2.5_O_4_ demonstrated higher thermal stability than NiCo_2_O_4_. After thermal treatment at 400 °C (Figure 11a), the DSC signal difference for Ni_0.5_Co_2.5_O_4_ before and after treatment is twice as low (0.15 μV mg^−1^ at 400 °C). The highest decrease in the DSC signal from 3.3 μV mg^−1^ to 3.1 μV mg^−1^ was also obtained at 450 °C after treatment at 450 °C (Figure 11b). The high susceptibility of Ni_x_Co_3−x_O_4_ materials to high temperatures agrees with earlier reports [27,28].

The fast deterioration of the initial activity in NiCo_2_O_4_ correlates with a strongly pronounced NiO segregation and its defective structure (Figure 7). Ni_0.5_Co_2.5_O_4_ contained fewer structural defects and no NiO segregation (Figure 8) but showed a slightly inhomogeneous Ni distribution inside hexagonal plates. The latter one can affect the catalyst’s stability at higher operation temperatures. This is because higher operation temperatures usually promote further phase segregation, causing catalyst destabilization and decreasing catalyst activity. Otherwise, the thermal stability of 2D materials may be limited compared to nanoparticles [37]. It is conceivable that the stability of Ni_x_Co_3−x_O_4_ catalysts could be further improved when Ni ions are completely incorporated into the structure of Co_3_O_4_. This could be achieved by using stabilizing additives or by applying alternative synthesis methods.

**Figure 12:**
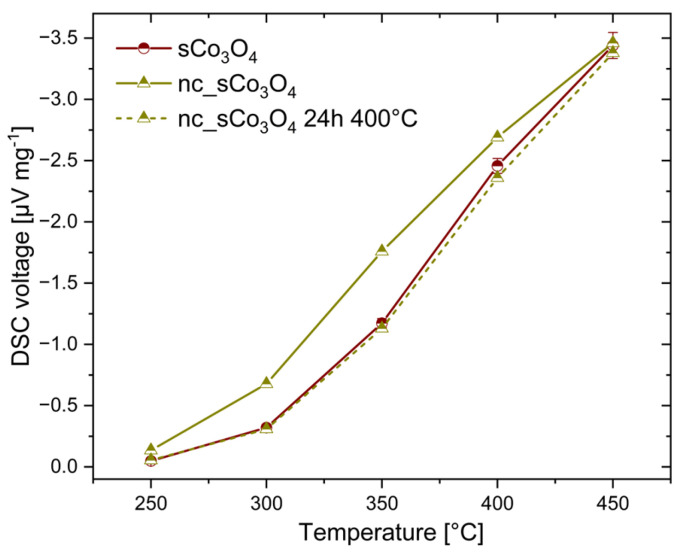
Effect of calcination temperature on the temperature-dependent DSC signal to 1% methane, demonstrated for sCo_3_O_4_.

In addition to the stability experiments performed at 400 °C and 450 °C, the impact of the calcination on the catalyst activity was examined. The most active sCo_3_O_4_ was chosen for the examination. All investigated catalysts were dried at 130 °C and subsequently calcined at 350 °C. To see the effect of calcination, sCo_3_O_4_ was additionally examined directly after drying without a calcination step (denoted as nc_sCo_3_O_4_). For further comparison, non-calcined sCo_3_O_4_ was then calcined at 400 °C for 24 h (denoted as nc_sCo_3_O_4_ 24 h 400 °C). Figure 12 shows the temperature-dependent DSC signals of original sCo_3_O_4_, nc_sCo_3_O_4_, and nc_sCo_3_O_4_ for 24 h at 400 °C.

nc_sCo_3_O_4_ revealed a considerably higher signal than sCo_3_O_4_, especially in the low-temperature range between 250 °C and 350 °C. The DSC signal at 350 °C increased from 1.8 μV mg^−1^ for nc_sCo_3_O_4_ to 1.2 μV mg^−1^ for sCo_3_O_4_. The nc_sCo_3_O_4_ sample calcined at 400 °C showed comparable activity to the original sCo_3_O_4_. Such an effect of calcination temperature on the activity of sCo_3_O_4_ demonstrates that a Co_3_O_4_-based catalyst features temperature-sensitive catalytic functionalities. Regarding the XRD investigations (Figure 2), it is obvious that higher temperatures cause a lower amount of active surface oxygen present on the surface of Co_3_O_4_ catalysts. Reduced surface oxygen as active species in the C–H bond dissociation has a negative effect on the catalytic activity of Co_3_O_4_ at low operation temperatures. Otherwise, catalyst calcination at temperatures higher than later operating temperatures is required to stabilize the structure of Co_3_O_4_ catalysts despite the associated reduced activity [38]. Nevertheless, the optimal operation temperature for Co_3_O_4_-based catalysts without precise metals is in the low-temperature range, below ≤350 °C.

The next step in the development of the metal-oxide catalysts for catalytic gas sensors is the examination of metal-decorated Co_3_O_4_ catalysts. Metal oxides offer several advantages over aluminum oxide as support material, such as stronger interactions with metal nanoparticles. Thus, the advantages and disadvantages of such kind of catalysts should be carefully examined. 

## 4. Conclusions

In conclusion, the investigations of the thermal response of Ni-doped and undoped Co_3_O_4_ metal oxides synthetized by co-precipitation towards 1% methane have shown that Ni doping improves the catalytic response of Co_3_O_4_. However, control of the structure and morphology of Co_3_O_4_ has a comparable impact on the activity and thermal response compared to doping, as was shown using sCo_3_O_4_ catalyst with optimized morphology. Moreover, the doping of the spinel structure of Co_3_O_4_ with nickel requires a balanced Ni:Co ratio and highly controllable synthesis conditions to reduce or eliminate the appearance of phase segregation, which is mainly responsible for the low thermal stability of Ni_x_Co_3−x_O_4_ catalysts. Because of its higher thermal stability, pure Co_3_O_4_ is preferable as a catalyst and for further modification with precise metals, which are applied to increase catalyst activity and sensor response at lower operation temperatures. Besides the detection of methane, Co_3_O_4_-based materials are of special interest for the detection of hydrogen since hydrogen can usually be detected at noticeably lower temperatures than hydrocarbons due to its significantly higher reactivity.

## Data Availability

Data are contained within the article and Supplementary Materials.

## Supplementary Materials

The following supporting information can be downloaded at: https://www.mdpi.com/article/10.3390/s24082599/s1, Figure S1: DSC signal of sCo_3_O_4_ catalyst measured in the temperature range between 250 and 450 °C.
