# Supplementary material for: Co3O4-Based Materials as Potential Catalysts for Methane Detection in Catalytic Gas Sensors
Source: Sensors (Basel). 2024 Apr 18;24(8):2599. doi: 10.3390/s24082599 (PMC11054299; doi:10.3390/s24082599)
Supplement: Supplementary file 1 [file sensors-24-02599-s001.zip › sensors-2898089-supplementary.pdf]

Supplementary Materials

Co_3O_4-based materials as potential catalysts for methane detection in catalytic gas sensors

Olena Yurchenko[1]*, Patrick Diehle[2], Frank Altmann[2], Katrin Schmitt[1,3] and Jürgen Wöllenstein [1,3]

[1] Fraunhofer Institute for Physical Measurement Techniques (IPM), Freiburg 79110, Germany
[2] Fraunhofer Institute for Microstructure of Materials and Systems (IMWS), Halle 06120, Germany
[3] Department of Microsystems Engineering (IMTEK), University of Freiburg, 79110 Freiburg, Germany
* Correspondence: olena.yurchenko@ipm.fraunhofer.de;

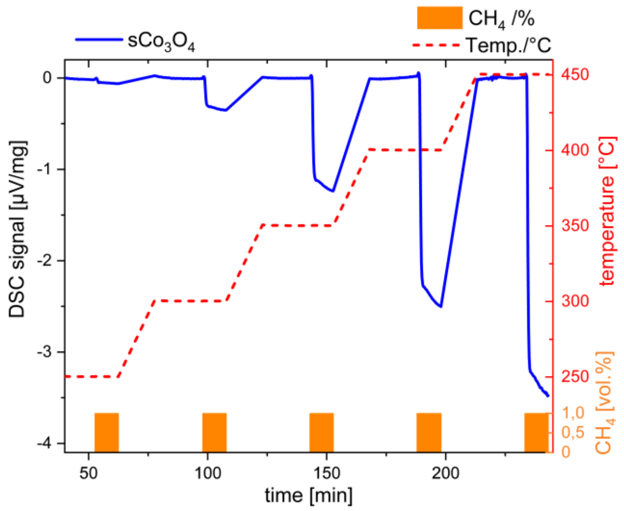

Figure S1. DSC signal of sCo_3O_4 catalyst measured in the temperature range between 250 and 450 °C.
